# Continuous low dose Thalidomide: a phase II study in advanced melanoma, renal cell, ovarian and breast cancer

**DOI:** 10.1054/bjoc.1999.1004

**Published:** 2000-02

**Authors:** T Eisen, C Boshoff, I Mak, F Sapunar, M M Vaughan, L Pyle, S R D Johnston, R Ahern, I E Smith, M E Gore

**Affiliations:** Department of Medicine, Royal Marsden Hospital, Fulham Road, London, SW3 6JJ, UK; Department of Clinical Neurophysiology, Chelsea and Westminster Hospital, London, SW10 9NH, UK

**Keywords:** Thalidomide, TNF-α, renal cell carcinoma

## Abstract

To grow and metastasize, solid tumours must develop their own blood supply by neo-angiogenesis. Thalidomide inhibits the processing of mRNA encoding peptide molecules including tumour necrosis factor-alpha (TNF-α) and the angiogenic factor vascular endothelial growth factor (VEGF). This study investigated the use of continuous low dose Thalidomide in patients with a variety of advanced malignancies. Sixty-six patients (37 women and 29 men; median age, 48 years; range 33–62 years) with advanced measurable cancer (19 ovarian, 18 renal, 17 melanoma, 12 breast cancer) received Thalidomide 100 mg orally every night until disease progression or unacceptable toxicity was encountered. Three of 18 patients with renal cancer showed partial responses and a further three patients experienced stabilization of their disease for up to 6 months. Although no objective responses were seen in the other tumour types, there were significant improvements in patients' sleeping (*P*< 0.05) and maintained appetite (*P*< 0.05). Serum and urine concentrations of basic fibroblast growth factor (bFGF), TNF-α and VEGF were measured during treatment and higher levels were associated with progressive disease. Thalidomide was well tolerated: Two patients developed WHO Grade 2 peripheral neuropathy and eight patients developed WHO grade 2 lethargy. No patients developed WHO grade 3 or 4 toxicity. Further studies evaluating the use of Thalidomide at higher doses as a single agent for advanced renal cancer and in combination with biochemotherapy regimens are warranted. © 2000 Cancer Research Campaign


*British Journal of Cancer* (2000) **82**(4), 812–817
© 2000 Cancer Research Campaign
DOI: 10.1054/ bjoc.1999.1004, available online at http://www.idealibrary.com on IDEAL®

### Continuous low dose Thalidomide: a phase II study in advanced melanoma, renal cell, ovarian and breast cancer

**T Eisen[1,*], C Boshoff[1,*], I Mak[2], F Sapunar[1], MM Vaughan[1], L Pyle[1], SRD Johnston[1], R Ahern[1], IE Smith[1] and ME Gore[1]**

[1]Department of Medicine, Royal Marsden Hospital, Fulham Road, London SW3 6JJ, UK; [2]Department of Clinical Neurophysiology, Chelsea and Westminster Hospital, London SW10 9NH, UK

**Summary** To grow and metastasize, solid tumours must develop their own blood supply by neo-angiogenesis. Thalidomide inhibits the processing of mRNA encoding peptide molecules including tumour necrosis factor-alpha (TNF-α) and the angiogenic factor vascular endothelial growth factor (VEGF). This study investigated the use of continuous low dose Thalidomide in patients with a variety of advanced malignancies. Sixty-six patients (37 women and 29 men; median age, 48 years; range 33–62 years) with advanced measurable cancer (19 ovarian, 18 renal, 17 melanoma, 12 breast cancer) received Thalidomide 100 mg orally every night until disease progression or unacceptable toxicity was encountered. Three of 18 patients with renal cancer showed partial responses and a further three patients experienced stabilization of their disease for up to 6 months. Although no objective responses were seen in the other tumour types, there were significant improvements in patients' sleeping ($P < 0.05$) and maintained appetite ($P < 0.05$). Serum and urine concentrations of basic fibroblast growth factor (bFGF), TNF-α and VEGF were measured during treatment and higher levels were associated with progressive disease. Thalidomide was well tolerated: Two patients developed WHO Grade 2 peripheral neuropathy and eight patients developed WHO grade 2 lethargy. No patients developed WHO grade 3 or 4 toxicity. Further studies evaluating the use of Thalidomide at higher doses as a single agent for advanced renal cancer and in combination with biochemotherapy regimens are warranted. © 2000 Cancer Research Campaign

**Keywords**: Thalidomide; TNF-α; renal cell carcinoma

Angiogenesis in melanoma and various carcinomas often correlates with the likelihood of the development of metastases and the prognosis of such patients (Weidner et al, 1991; Folkman, 1995*b*; Weidner, 1995; Erhard et al, 1997; Schiffenbauer et al, 1997). A number of different angiogenic and anti-angiogenic factors are known to be involved in regulation of the angiogenic cascade, including vascular endothelial growth factor (VEGF), acidic and basic fibroblast growth factor (bFGF), hepatocyte growth factor (scatter factor), platelet-derived growth factor, transforming growth factor-β1, interleukin-8 (IL-8), tumour necrosis factor-α (TNF-α), SPARC peptides, angiostatin and interferon-α (Bussolino et al, 1996). These and other cytokines produced by endothelial cells can stimulate tumour cells in a paracrine manner (Hamada et al, 1992). Furthermore, normal endothelial cells within the tumour mass may be abnormally stimulated by diffusible growth factors released by tumour cells (Folkman, 1995*a*).

Angiogenesis inhibitors are now under investigation in patients (Burrows and Thorpe, 1994; Folkman, 1995*b*; Ziegler, 1996). These include the commercially available agents Thalidomide, interferon-α, paclitaxel, tamoxifen and medroxyprogesterone acetate, and experimental agents including neutralizing humanized antibodies to VEGF and bFGF, metalloproteinase inhibitors, TMP-470 (a synthetic analogue of fumagillin) and IL-12 (Ingber et al, 1990; D'Amato et al, 1994; Voest et al, 1995; Gasparini, 1996; Talbot and Brown, 1996). Anti-angiogenic therapy appears to have its optimum efficacy if given daily or intermittently over a long period, acts mainly on proliferating capillary endothelial cells, is generally of low toxicity and, in long-term animal studies, drug resistance has not developed (Folkman, 1995*b*).

Thalidomide is known to have powerful anti-angiogenic activity (D'Amato et al, 1994; Battegay, 1995), can eradicate experimental tumours in mice (Ching et al, 1995) and is used to treat the vascular tumour Kaposi's sarcoma with 60% of patients responding or achieving stable disease (Ziegler, 1996). Non-malignant indications for Thalidomide include retinal neo-vascularization due to macular degeneration, leprosy, Crohn's disease, pulmonary tuberculosis, recurrent graft-versus-host disease and human immuno-deficiency virus-related oral aphthous ulcers (Vogelsang et al, 1992; Asscher, 1994; Crawford, 1994; Folkman, 1995*a*; Tramontana et al, 1995; Jacobson et al, 1997; Wettstein and Meagher, 1997). The notorious teratogenic effects of Thalidomide when administered to women in the first trimester of pregnancy in the 1960s (McBride, 1961; Lenz, 1962) have been attributed to inhibition of blood vessel growth in the developing fetal limb bud (D'Amato et al, 1994) and more recently to free radical-mediated oxidative DNA damage (Parman et al, 1999).

Thalidomide increases the degradation of the mRNA of a number of peptide-signalling molecules such as FGF (D'Amato et al, 1994) and TNF-α (Ching et al, 1995). The suppression of TNF-

*Received 30 June 1999*
*Revised 22 August 1999*
*Accepted 20 October 1999*

*Correspondence to:* ME Gore

*Present address: Department of Oncology, University College London, 91 Riding House Street, London W1P 8BT, UK

α in cancer patients may be of particular palliative benefit since high levels of TNF-α have previously been linked to cachexia and tumour-related malaise (Yoneda et al, 1991; Haslett, 1998).

The present phase II study assessed the efficacy and toxicity of continuous Thalidomide in the treatment of patients with metastatic melanoma, renal cell carcinoma and ovarian and breast cancer. In view of the dose-related peripheral sensory neuropathy found in patients on long-term Thalidomide, we chose to employ the low dose of Thalidomide 100 mg orally every night in this group of patients, many of whom had previously received neurotoxic chemotherapy, and to follow all patients with electrophysiological studies before and during therapy (Fullerton and Kremer, 1961; Powell and Gardner-Medwin, 1994). Although lower than most doses employed for shorter periods, experience from treating Behçet's syndrome suggests that a Thalidomide dose as low as 25 mg daily is clinically active (Eisenbud et al, 1987). As well as recording the palliative benefits and objective clinical responses to Thalidomide, we assessed biological responses by serially measuring serum TNF-α and serum and urinary VEGF and bFGF before and during treatment.

### MATERIALS AND METHODS

### Patients

Patients with histologically confirmed metastatic renal cell carcinoma, melanoma, ovarian cancer or breast cancer, were eligible for entry into this study. Eligibility criteria included the presence of metastatic disease that was measurable in at least one diameter greater than 1 cm by clinical examination or imaging. The disease must have shown progression between two recorded time points and be defined clinically or by imaging. Patients must not have received endocrine therapy, radiotherapy, biological agents or chemotherapy in the previous 4 weeks. Marker lesions must not have received radiotherapy at any time. Patients had to be of ECOG performance status 0–2. All patients had to be older than 18 years and had a life expectancy of greater than 12 weeks. Patients required adequate bone marrow reserve with white blood count greater than $3 \times 10^9$ $l^{-1}$, platelets greater than $100 \times 10^9$ $l^{-1}$, and haemoglobin more than 10 g $dl^{-1}$, normal renal function or a glomerular filtration rate greater than 60 ml $min^{-1}$ as assessed by EDTA clearance and adequate hepatic function defined as liver function tests less than twice the normal values, unless due to metastatic disease. Female patients had to have a negative pregnancy test. Any women of child-bearing age had to use adequate contraceptive methods during the study. In view of the anti-angiogenic action of Thalidomide, any wound must have healed and any surgery other than a skin biopsy must have been performed more than 4 weeks prior to study entry. Patients with pre-existing peripheral neuropathy of WHO grade 2 or greater were not eligible for the study.

The protocol was approved by the Royal Marsden Hospital Ethics Committee and all patients gave written informed consent.

### Treatment

Following baseline assessment at study entry, patients started taking Thalidomide 100 mg (Penn Pharmaceuticals Ltd, Gwent, UK) orally every night. Treatment continued until there was evidence of disease progression or unacceptable toxicity was encountered, or the patient wished to stop treatment for any reason.

### Assessment of response and toxicity

All patients were examined clinically before treatment. Baseline laboratory investigations included full blood count, serum biochemistry, sensory nerve action potential (SNAP), serum and urine were frozen and stored for bFGF, VEGF and TNF-α assessment by enzyme-linked immunosorbent assay (ELISA) (R&D Systems, Abingdon, UK), electrocardiogram and serum pregnancy test for women of child-bearing age. Patients underwent imaging or clinical measurement as appropriate to assess their disease status at the start of treatment. Patients completed a symptom distress scale (HAD and Rotterdam scores) before starting treatment.

Patients had a clinical examination, full blood count and biochemistry every month whilst on treatment. Patients received a SNAP test, serum and urine were frozen and stored for bFGF, VEGF and TNF-α assessment, clinical imaging, symptom distress scale questionnaire and toxicity evaluation at 1, 3, 6, 9 and 12 months while on treatment.

Response was assessed according to standard International Union Against Cancer criteria following clinical measurement or imaging. Toxicity was assessed according to WHO criteria at each assessment visit (Millar et al, 1981). Patients who received at least 4 weeks of treatment were assessable for response and all patients were assessable for toxicity. The response duration was defined as the time elapsed between start of treatment with Thalidomide and the date of progressive disease or last follow-up evaluation. Stable disease was defined as where neither partial response nor progressive disease could be established.

### Statistical considerations

The threshold response rate for each individual tumour type below which the treatment would be considered ineffective was 15%. It was originally intended to recruit 19 patients for each tumour type. If there were no responses in these patients the study would be terminated. There was only a 5% chance that this would occur if the true response rate was 15%. We decided to terminate the study before full recruitment had been completed because of the responses seen and because of slow recruitment of patients with breast cancer. The $\chi^2$ test and McNemar test for trend were used to assess differences in quality of life before and during treatment with Thalidomide.

### RESULTS

### Patient characteristics

Between July 1997 and March 1998, 66 eligible patients with advanced solid tumours under the care of the Royal Marsden Hospital, London and Sutton, were entered into the study; 19 patients had ovarian cancer, 13 men and five women had renal carcinoma, 12 men and five women had melanoma and 12 women had breast cancer (Table 1). Two other patients with melanoma were also entered but were found not to be eligible because, on review of radiological imaging, they showed a late response to biochemotherapy given prior to entering the study. The median age of patients was 48 years, range 33–62 years. The majority of patients were fit, with ten having a performance status of 0 and 38 having a performance status of 1. However, 18 patients had a performance status of 2. Many of the patients had received multiple treatments before entering the study.

*© 2000 Cancer Research Campaign*

**Table 1** Patient characteristics

| | |
|---|---|
| Number of patients | 66 |
| Ovary | 19 |
| Renal | 18 |
| Melanoma | 17 |
| Breast | 12 |
| Male | 29 |
| Female | 37 |
| Age (years) | |
| Median | 48 |
| Range | (33–62) |
| Performance status | |
| 0 | 10 |
| 1 | 38 |
| 2 | 18 |
| Previous treatment | |
| Chemotherapy | 44 |
| Radiotherapy | 24 |
| Surgery | 22 |
| Hormone | 14 |
| Immuno-chemotherapy | 10 |
| Immunotherapy | 4 |
| None | 10 |

### Tumour response

All 66 eligible patients entered into the study were evaluable for response and toxicity (Table 2). Three partial responses were seen in the 18 patients treated for renal carcinoma. One response lasted 5 months, the other two partial responses are continuing at 5 and 11 months of follow-up. The response continuing at 11 months was dramatic and is illustrated in Figure 1. This patient had progressed rapidly on biochemotherapy with neck lymphadenopathy and symptomatic lung metastases and had severe malaise before starting Thalidomide. Within 24 h of starting Thalidomide his malaise had resolved and his performance status had improved to 0. Over the next 5 months his lung and lymph node metastases shrunk and a bronchoscopic biopsy of the largest lung metastasis at 11 months showed necrotic tumour only. It was also noticeable in the group with renal carcinoma that 13 patients experienced stabilization of their previously progressive disease: three of these patients had stable disease for 3 months or longer, whilst the other ten patients had stable disease for only 1–3 months.

Of the remaining 48 patients with melanoma, ovarian carcinoma or breast carcinoma none showed any objective tumour response to treatment with Thalidomide. However, three patients had a differential response and one patient with rapidly progressive skin deposits of melanoma on his leg experienced symptomatic improvement and was able to walk more than 100 yards, having previously been chair-bound.

**Table 2** Responses to Thalidomide

| | No. of patients | Partial response | Stable disease (> 3 months) |
|---|---|---|---|
| Ovary | 19 | 0 (0%) | 1 (5%) |
| Renal | 18 | 3 (17%) | 3 (17%) |
| Melanoma | 17 | 0 (0%) | 1 (6%) |
| Breast | 12 | 0 (0%) | 0 (0%) |

**Table 3** Concentration of angiogenic markers in serum and urine before and during Thalidomide therapy (pg ml$^{-1}$)

| Serum | Controls $n$ = 2 | | Pre-Thalidomide $n$ = 41 | | On Thalidomide (4–6 weeks of treatment) $n$ = 23 | |
|---|---|---|---|---|---|---|
| | Mean | Range | Mean | Range | Mean | Range |
| VEGF | 51 | 27–74 | 568 | 114–1861 | 554 | 110–1080 |
| bFGF | < 10 | < 10 | < 10 | < 10–28 | < 10 | < 10–20 |
| TNF-α | < 10 | < 10 | < 10 | < 10–16 | < 10 | < 10–20 |
| **Urine** | Controls $n$ = 2 | | Pre-Thalidomide $n$ = 31 | | On Thalidomide (4–6 weeks of treatment) $n$ = 13 | |
| | Mean | Range | Mean | Range | Mean | Range |
| VEGF | 83 | 30–145 | 106 | 24–695 | 222 | 10–1000 |
| bFGF | < 10 | < 10 | < 10 | < 10 | < 10 | < 10 |
| TNF-α | < 10 | < 10 | < 10 | < 10 | < 10 | < 10 |

### Angiogenic markers

A wide range of serum and urinary VEGF levels was found in patients and in two controls (Table 3). No clear relationship between the absolute level of VEGF and tumour response was found, nor were there any clear differences between different tumour types. However, a rising VEGF level was associated with progressive disease in six of 11 patients who had serial measurements.

bFGF was only detected in the serum of 12 patients all of whom had early progressive disease. bFGF and free TNF-α were not detected in the urine of any patient. Free TNF-α was detected in the serum of one patient with melanoma, one patient with breast cancer and one patient with ovarian cancer.

### Toxicity and symptomatic side-effects

Thalidomide 100 mg orally every night was generally very well tolerated with no WHO grade 3 or 4 toxicities (Table 4). The main toxicity was lethargy (38 patients grade 1 and eight patients grade 2). Grade 2 peripheral neuropathy was detected clinically in two patients who had been on treatment for 11 months. On interrupting Thalidomide, the neuropathy resolved and treatment recommenced at 50 mg each night. All patients had SNAP tests and no other patient developed sensory neuropathy. One patient developed a WHO grade 2 headache shortly after starting Thalidomide and another developed WHO grade 2 peripheral oedema. Both side-effects resolved on stopping Thalidomide. Ten patients developed WHO grade 1 constipation and two developed an itchy WHO grade 1 skin rash. These side-effects responded to standard measures.

**Table 4** Symptomatic side-effects

| | WHO grade 1 $n$ | (%) | WHO grade 2 $n$ | (%) |
|---|---|---|---|---|
| Lethargy | 38 | 58 | 8 | 12 |
| Peripheral neuropathy | 0 | | 2 | 3 |
| Headache | 0 | | 1 | 2 |
| Oedema | 0 | | 1 | 2 |
| Constipation | 10 | 15 | 0 | |
| Skin rash | 2 | 3 | 0 | |

 *© 2000 Cancer Research Campaign*

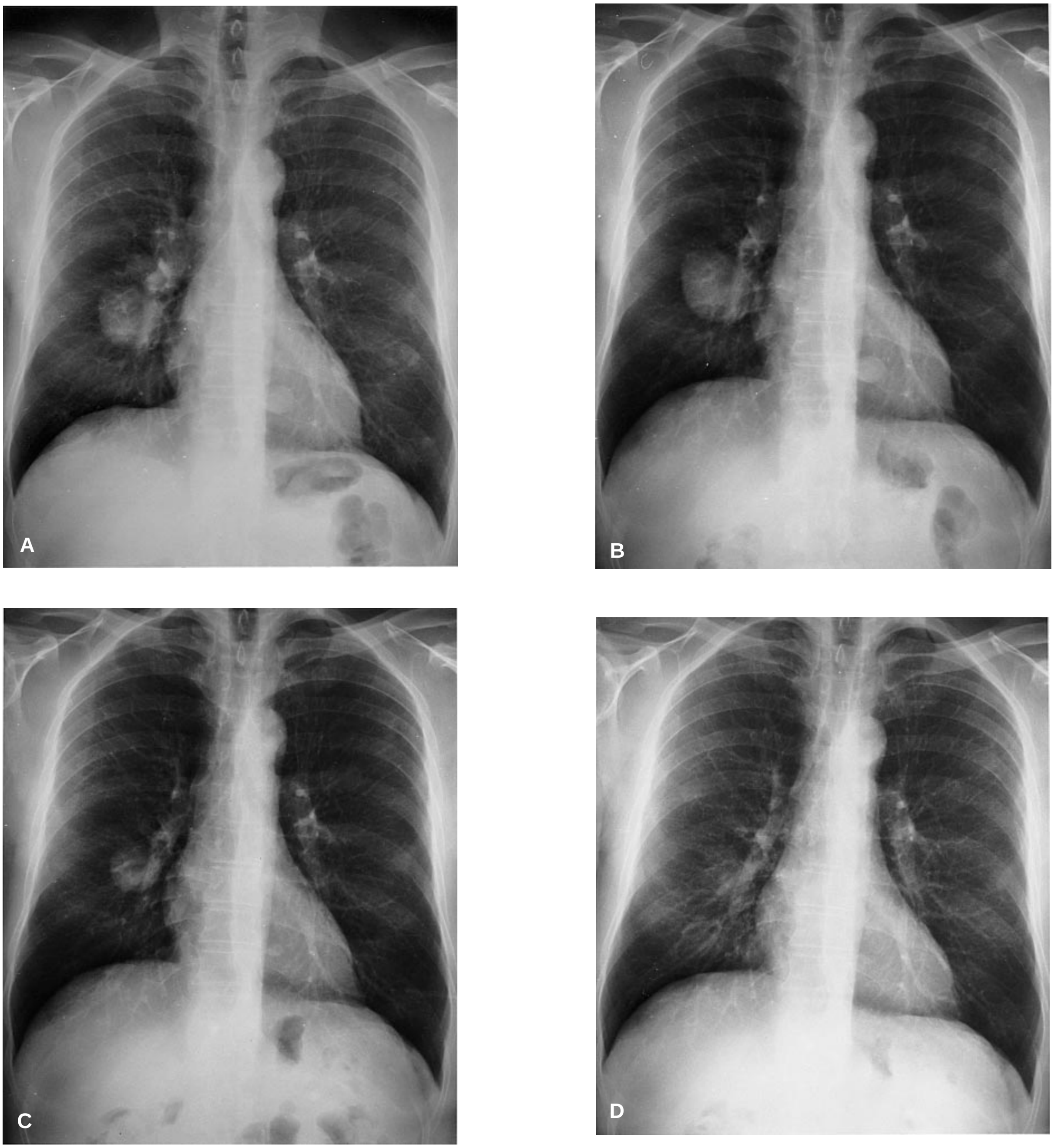

**Figure 1** Chest X-ray series illustrating response to Thalidomide after rapid progression on biochemotherapy. (**A**) At end of biochemotherapy. (**B**) At start of Thalidomide 6 weeks later, showing progressive disease. (**C**) After 2 months of Thalidomide showing PR. (**D**) After 4 months of Thalidomide showing further PR

Of 15 patients who completed serial HAD and Rotterdam scores, 11 experienced an improvement in sleeping and 14 experienced a maintained or improved appetite. Both of these findings are statistically significant ($P < 0.05$), although based on a very small number of patients.

### DISCUSSION

Despite employing a low dose of Thalidomide, we observed encouraging responses in patients with renal cell carcinoma. In 18 patients treated for renal carcinoma, three showed partial responses to Thalidomide and 13 showed stable disease, three of them for more than 3 months. Two of the three responding patients had progressed on previous biochemotherapy with interferon-α, 5-fluorouracil and IL-2. Late responses to immunotherapy are seen both in renal cell carcinoma and in melanoma. However, the partial responses in these two patients are unlikely to be due to a late response to biochemotherapy for three reasons. First, patients were found to have rapidly progressive disease both during and after biochemotherapy with disease appearing in previously

*© 2000 Cancer Research Campaign* 

unaffected sites. Secondly, the patients obtained significant palliative benefit within 24 h of starting Thalidomide and thirdly, objective tumour shrinkage started within 2 weeks of starting Thalidomide.

We also observed stable disease in 13 of the remaining 15 patients with renal cell carcinoma. In three of these patients the stable disease was observed for more than 3 months. These findings may be important as the patients had aggressive and advanced disease. Four patients with melanoma had stable disease for periods of up to 5 months and one patient with melanoma experienced significant symptomatic relief from his melanomatous leg deposits. However, no objective responses were observed in any of the other patients. Patients found repeated quality of life assessments irksome and this accounts for the poor uptake. The significant symptomatic benefits, including improved sleep and appetite, could be a result of self-selection by those completing the questionnaires. However, the palliative benefits were credible and indeed possibly predictable in view of the known sedative and appetite-enhancing effects of Thalidomide (Powell and Gardner-Medwin, 1994).

Thalidomide could act either by inhibiting angiogenesis or by inhibiting factors secreted by the tumour. A humoural basis for the action of Thalidomide is suggested by the rapid onset of benefit in our responding patients. The most likely candidate target in renal cell carcinoma is TNF-α. This cytokine is known to be secreted by renal cell carcinomas (Mizutani et al, 1994), enhances neo-angiogenesis (Fajardo et al, 1992), augments the stimulation of renal carcinoma cells by IL-6 (Koo et al, 1992) and contributes to many of the systemic symptoms of advanced malignancy (Yoneda et al., 1991; Haslett, 1998). The inhibition of TNF-α by Thalidomide may therefore be of particular interest in renal cell carcinoma.

No clear effect of Thalidomide was observed in serial measurements of serum and urine TNF-α, VEGF and bFGF. Free TNF-α was only detected in the serum of three patients at the time that their progressive disease was noted. Serum TNF-α levels may therefore be of limited value in the selection of patients for Thalidomide treatment or the monitoring of their response. In future studies we will monitor concentrations of TNF-α in fresh plasma samples which may be more reliable. The significance of systemic levels of TNF-α in renal cell carcinoma requires further investigation. It is possible that local concentrations in the tumour bed are of greater significance. We found a wide range of serum and urinary levels of VEGF both in patients and in two controls. In 11 patients who had serial concentrations measured, a rising VEGF level was found in all six patients with progressive disease. Similarly, bFGF was only detected in the serum of 12 patients, all of whom progressed early.

Thalidomide 100 mg orally each night was well tolerated for at least 11 months even in patients who have previously been treated with neurotoxic chemotherapy. Two patients who have received Thalidomide for 11 months and 5 months have developed grade 2 peripheral neuropathy which resolved on interrupting treatment. Many patients noticed mild lethargy and constipation but none developed grade 3 or 4 toxicities.

Our findings suggest that Thalidomide may be useful in the management of advanced renal carcinoma and possibly of symptomatic benefit in other solid malignancies. This warrants further studies, particularly at a higher dose in patients with renal carcinoma. A fuller appreciation of the effect of Thalidomide on TNF-α levels is needed because Thalidomide may be of particular benefit to patients who express high levels of TNF-α. Such a correlation would allow better selection of patients who may benefit from this and other anti-angiogenic therapies. Finally, the use of Thalidomide in combination with other treatments should be investigated. Highest response rates in renal cell carcinoma are reported with a combination of interferon-α, 5-fluorouracil and IL-2 (Lopez-Hanninen et al, 1996). The considerable side-effects may be related to the finding that IL-2 induces high levels of TNF-α (Weidmann et al, 1992). The increased levels of TNF-α are unlikely to contribute to the beneficial effects of this treatment, since previous experience with TNF-α in renal carcinoma has shown severe side-effects with a poor response rate (reviewed by Boshoff and Jones, 1996). Moreover, these high levels of TNF-α may also stimulate growth of renal carcinoma cells (Lopez-Hanninen et al, 1996). Interestingly, two of the three patients who had partial responses to Thalidomide had progressed rapidly on biochemotherapy for their advanced renal carcinoma. It is tempting to speculate that these patients' tumours were responsive to stimulation with TNF-α and therefore grew rapidly on biochemotherapy and responded to inhibition of TNF-α by Thalidomide. There may be a rationale for combining Thalidomide with biochemotherapy, as a possible way both of reducing side-effects and increasing the efficacy of treatment for renal cell carcinoma.

### ACKNOWLEDGEMENTS

The authors would like to thank Jackie Reeves and Graham Simmons for carrying out the ELISA for bFGF, VEGF and TNF-α, Liz Miller and Fiona Ramage for data collection and Chris Bunker for useful discussions.

### REFERENCES

Asscher W (1994) Safety of Thalidomide. *Br Med J* 309: 193–194 (letter)

Battegay EJ (1995) Angiogenesis: mechanistic insights, neovascular diseases, and therapeutic prospects. *J Mol Med* **73**: 333–346

Boshoff C and Jones AL (1996) Tumour necrosis factor. In: *Immunotherapy in Cancer*, Gore ME and Riches P (eds), pp. 77–103. John Wiley: London

Burrows FJ and Thorpe PE (1994) Vascular targeting – a new approach to the therapy of solid tumours. *Pharmacol Ther* **64**: 155–174

Bussolino F, Albini A, Camussi G, Presta M, Vigliotto G, Ziche M and Persico G (1996) Role of soluble mediators in angiogenesis. *Eur J Cancer* (Special issue: basic and clinical research on angiogenesis) **32A**: 2401–2412

Ching LM, Xu ZF, Gummer BH, Palmer BD, Joseph WR and Baguley BC (1995) Effect of Thalidomide on tumour necrosis factor production and anti-tumour activity induced by 5,6-dimethylxantheone-4-acetic acid. *Br J Cancer* **72**: 339–343

Crawford CL (1994) Use of Thalidomide in leprosy. *Adverse Drug React Toxicol Rev* **13**: 177–192

D'Amato RJ, Loughnan MS, Flynn E and Folkman J (1994) Thalidomide is an inhibitor of angiogenesis. *Proc Natl Acad Sci USA* **91**: 4082–4085

Eisenbud L, Horowitz I and Kay B (1987) Recurrent aphthous stomatitis of the Behçet's type: successful treatment with Thalidomide. *Oral Surg Oral Med Oral Pathol* **64**: 289–292

Erhard H, Rietveld FJ, van Altena MC, Brocker EB, Ruiter DJ and de Waal RM (1997) Transition of horizontal to vertical growth phase melanoma is accompanied by induction of vascular endothelial growth factor expression and angiogenesis. *Melanoma Res* Suppl **2**: S19–26

Fajardo LF, Kwan HH, Kowalski J, Prionas SD and Allison AC (1992) Dual role of tumor necrosis factor-alpha in angiogenesis. *Am J Pathol* 140: 539–544

Folkman J (1995*a*) Angiogenesis in cancer, vascular, rheumatoid and other disease. *Nat Med* **1**: 27–31

Folkman J (1995*b*) Clinical applications of research on angiogenesis. *N Engl J Med* **333**: 1757–1763

Fullerton PM and Kremer M (1961) Neuropathy after intake of Thalidomide (Distaval). *Br Med J* **2**: 853–858

© 2000 Cancer Research Campaign

Gasparini G (1996) Clinical significance of the determination of angiogenesis in human breast cancer: update of the biological background and overview of the Vicenza studies. *Eur J Cancer* **32A**: 2485–2493

Hamada J, Cavanaugh PG, Lotan O and Nicolson GL (1992) Separable growth and migration factors for large cell lymphoma cells secreted by microvascular endothelial cells derived from target organs for metastasis. *Br J Cancer* **66**: 349–354

Haslett PA (1998) Anticytokine approaches to the treatment of anorexia and cachexia. *Semin Oncol* **25**: 53–57

Ingber D, Fujita T, Kishimoto S, Sudo K, Kanamuru T, Brem H and Folkman J (1990) Synthetic analogues of fumagillin that inhibit angiogenesis and suppress tumour growth. *Nature* **348**: 555–557

Jacobson JM, Greenspan JS, Spritzler J, Ketter N, Fahey JL, Jackson JB, Fox L, Chernoff M, Wu AW, MacPhail LA, Vasquez GJ and Wohl DA (1997) Thalidomide for the treatment of oral aphthous ulcers in patients with human immunodeficiency virus infection. *N Engl J Med* **336**: 1487–1493

Koo AS, Armstrong C, Bochner B, Shimabukuro T, Tso CL, de Kernion JB and Belldegrum A (1992) Interleukin-6 and renal cell cancer: production, regulation and growth effects. *Cancer Immunol Immunother* **35**: 97–105

Lenz W (1962) Thalidomide and congenital abnormalities. *Lancet* **1**: 45 (letter)

Lopez-Hanninen E, Kirchner H and Atzpodien J (1996) Interleukin-2 based home therapy of metastatic renal cell carcinoma: risks and benefits in 215 consecutive single institution patients. *J Urol* **155**: 19–25

McBride WG (1961) Thalidomide and congenital abnormalities. *Lancet* **2**: 1358 (letter)

Millar AB, Hoogstraten B and Staquet M (1981) Reporting results of cancer treatment. *Cancer* **47**: 207–214

Mizutani Y, Bonavida B, Nio Y and Yoshida O (1994) Overcoming TNF-alpha and drug resistance of human renal cell carcinoma cells by treatment with pentoxifylline in combination with TNF-alpha or drugs: the role of TNF-alpha downregulation in tumor cell sensitization. *J Urol* **151**: 1697–1702

Parman T, Wiley MJ and Wells PG (1999) Free radical-mediated oxidative DNA damage in the mechanism of Thalidomide teratogenicity. *Nat Med* **5**: 582–585

Powell RJ and Gardner-Medwin JM (1994) Guideline for the clinical use and dispensing of Thalidomide. *Postgrad Med J* **70**: 901–904

Schiffenbauer YS, Abramovitch R, Meir G, Nevo N, Holzinger M, Itin A, Keshet E and Neeman M (1997) Loss of ovarian function promotes angiogenesis in human ovarian carcinoma. *Proc Natl Acad Sci USA* **94**: 13203–13208

Talbot DC and Brown PD (1996) Experimental and clinical studies on the use of matrix metalloproteinase inhibitors for the treatment of cancer. *Eur J Cancer* **32A**: 2528–2533

Tramontana JM, Utaipat U, Molloy A, Akarasewi P, Burroughs M, Makonkawkeyoon S, Johnson B, Klausner JD, Rom W and Kaplam G (1995) Thalidomide treatment reduces tumour necrosis alpha production and enhances weight gain in patients with pulmonary tuberculosis. *Mol Med* **1**: 384–397

Voest EE, Kenyon BM, O'Reilly MS, Truitt G, D'Amato RJ and Folkman J (1995) Inhibition of angiogenesis in vivo by interleukin 12. *J Natl Cancer Inst* **87**: 581–586

Vogelsang GB, Farmer ER, Hess AD, Altamonte V, Beschorner WE, Jabs DA, Corio RL, Levin LS, Colvin OM and Wingard JR (1992) Thalidomide for the treatment of chronic graft-versus-host disease. *N Engl J Med* **326**: 1055–1058

Weidmann E, Bergmann L, Stock J, Kirsten R and Mitrou PS (1992) Rapid cytokine release in cancer patients treated with interleukin-2. *J Immunother* 12: 123–131

Weidner N (1995) Intratumor microvessel density as a prognostic factor in cancer. *Am J Pathol* **147**: 9–19

Weidner N, Semple JP, Welch WR and Folkman J (1991) Tumor angiogenesis and metastasis – correlation in invasive breast cancer. *N Engl J Med* **324**: 1–8

Wettstein AR and Meagher AP (1997) Thalidomide in Crohn's disease. *Lancet* **350**: 1445–1446 (letter)

Yoneda T, Alsina MA, Chavez JB, Bonewald L, Nishimura R and Mundy GR (1991) Evidence that tumour necrosis factor plays a pathogenetic role in the paraneoplastic syndromes of cachexia, hypercalcaemia, and leukocytosis in a human tumour in nude mice. *J Clin Invest* **87**: 977–985

Ziegler J (1996) Angiogenesis research enjoys growth spurt in the 1990s. *J Natl Cancer Inst* **88**: 786–788

*© 2000 Cancer Research Campaign*
